# Effect of perceived stigma on work and social roles among individuals with mental health disorders in Saudi Arabia: findings from a national survey

**DOI:** 10.1186/s12991-023-00482-x

**Published:** 2023-12-14

**Authors:** Areej AlFattani, Lisa Bilal, Sami Y. Saad, Mohammad Talal Naseem, Sanaa Hyder, Abdulhamid Alhabib, Abdullah Alsubaie, Yasmin Altwaijri

**Affiliations:** 1 Biostatistics, Epidemiology and Scientific Computing Department, King Faisal Specialist Hospital and Research Centre, MBC - 03, P.O. Box 3354, 11211 Riyadh, Saudi Arabia; 2 King Salman Center for Disability Research, 12512 Riyadh, Saudi Arabia; 3 College of Medicine, SABIC Psychological Health Research & Applications Chair (SPHRAC), King Saud University, Riyadh, Saudi Arabia; 4 King Abdullah Medical City, Makkah, Saudi Arabia; 5 National Center for Mental Health Promotion, Ministry of Health, 11525 Riyadh, Saudi Arabia; 6 Edrak Medical Center, 12281 Riyadh, Saudi Arabia

**Keywords:** Stigma, Mental health, Saudi Arabia, Perceived, Survey, Social role

## Abstract

**Background:**

It is known worldwide that stigma towards mental illness exists. Studies on stigma perceived by patients with mental illness have shown decreased quality of life and a negative impact on work, school and social life. The aim of this study was to estimate the prevalence of perceived stigma among respondents who had been diagnosed with a mental illness during the past 12 months, its association with socio-demographic variables and its effect on work and social roles limitations among Saudis.

**Methods:**

The Saudi National Mental Health Survey (SNMHS) data were used for the analysis. The SNMHS is a nationally representative survey that was conducted using face-to -face interviews with Saudi individuals (age 15–65) in their households. Respondents were diagnosed (*N* = 639) with mental disorders based on a well-validated questionnaire—the Composite International Diagnostic Interview (CIDI) 3.0. Two dimensions from CIDI assessed perceived stigma: embarrassment and perceived discrimination.

**Results:**

The prevalence of perceived stigma was 27.8% among mentally ill respondents. Stigma was lower among respondents who didn’t seek any type of treatment than those who sought treatment OR = 0.28 (95% CI 0.084–0.935, *P* = 0.03). Respondents who reported perceived stigma had more work role limitations (OR = 1.1 95% CI 1.01–0.10 *P* 0.006) and social limitations (OR = 1.3 95% CI 0.99–1.62 *P* 0.05) than respondents who didn’t report stigma.

**Conclusion:**

Perceived stigma is experienced by mentally ill individuals and it negatively affects their work and social roles. Awareness programs to remove stigma and educate the public are needed to be established by policymakers and healthcare providers in Saudi Arabia.

## Background

Mental health disorders account for a significant proportion of the overall disease burden worldwide [1]. In the Global Burden of Disease Study 2016, mental and substance use disorders largely contributed to disability-adjusted life years (DALYs) worldwide in both sexes and in high socio-demographic countries [2]. Mental disorders contribute to 4.5% of DALYs in the Middle East and account for the ninth leading cause of disease burden [3]. However, despite these warning figures the public still negatively perceives the mentally ill more than the physically ill [4].

Studies have shown that stigma towards people with mental health disorders is a universal phenomenon and should be measured by culturally adapted tools [5]. A cross sectional study in 27 countries among schizophrenia patients using the Discrimination and Stigma Scale (DISC-12) reported that 47% had experienced negative discrimination in keeping or making friends and 64% had expected a discrimination behavior when applying for work and education opportunities [6]. Stigma can take many manifestations in different cultures like discrimination, limitations in social acceptance, prejudice and ignorance [7].

Results from the World Health Organization (WHO)-World Mental health (WMH) Survey Initiative with cross-national studies across 15 countries show that stigma concerning mental health is more apparent among lower education levels and lower socioeconomic societies as well as among ethnic minorities living in high-income countries [8]. Other studies on stigma perceived by patients with mental illness have shown decreased quality of life and a negative impact on work, school and social life [7–9].

Moreover, stigma is considered to be the highest ranked barrier for help-seeking [10, 11], and is highly associated with decreased self-esteem [12] and decreased treatment adherence [13]. According to a study’s findings from 2017, about 85% of people with serious mental disorders from low and intermediate-income countries do not receive the required treatment [14].

Although stigma towards mental health has about six subtypes [15], it is generally divided into three main types: structural stigma, public (or so-called social stigma) and self-stigma. Structural stigma defined as the rules, policies, and practices of social institutions that arbitrarily restrict the rights of, and opportunities for, people with mental illnesses [13]. While, social stigma refers to negative behavior towards individuals with mental illness. This appears as discrimination towards patients with mental illness, where they are seen as different or disqualified for certain privileges [16]. The consequences of this type of social stigma are numerous, such as unemployment and social isolation. On the other hand, self or perceived stigma is the devaluation, shame, secrecy and withdrawal triggered by applying negative stereotypes to oneself [17]. This can cause low self-esteem, shame and hopelessness [18]. On the other hand, Self-stigma according to healthcare providers has been identified as a factor in patients reluctant to seek consultation. In addition, it may even worsen due to social media-related information inaccuracies, exaggerations, or misinformation [19]. Such reports of stigma and its effect on delaying seeking help have been observed among Arab populations [20, 21]. There are earlier reports about tools to measure social stigma and others to measure self-stigma. The International Study of Discrimination and Stigma Outcomes network (INDIGO) created The Discrimination and Stigma Scale (DISC) 35-itmes to measure mental illness-related discrimination. More than 216 research users in 55 countries worldwide have accessed the scale [22]. Later on Brohan et al. developed The Discrimination and Stigma Scale Ultra Short (DISCUS) 11-items a reliable and valid unidimensional measure of experienced discrimination, suitable for clinical and research settings [23]. In these scales and others, embarrassment and discrimination (along with other negative behaviors) are considered important components of perceived of stigma [24]. These two indicators are among the recommended and widely used measures as per a large review of stigma measurement tools [25].

In the Kingdom of Saudi Arabia (KSA), the Saudi National Mental Health Survey (SNMHS) was recently conducted as part of the WMH Survey Initiative, a nationally representative survey of the general population in the KSA. The study showed that 22.3% of the Saudi population had at least one 12-month mental disorder using DSM-IV/CIDI 3.0 [26]. Moreover, only 13.9% of respondents with a 12‐month mental disorder obtained treatment, indicating high unmet need for treatment of mental disorders in KSA [27, 28].

Another study from KSA showed that over 50% of people will hide their mental disorders to avoid potential stigma [29]. However, other findings suggest that 36.5% of the general population agreed that they can speak to any person with mental illness, 43.5% don’t feel afraid when dealing with persons with mental illnesses, and 41.4% don’t refuse to sit with a person with mental illness [30]. However, there is a gap of knowledge in regards to the prevalence of stigma at a national level and its effect on social and work roles of individuals with mental illness in Saudi Arabia. Using the SNMHS data, this paper aims to assess the prevalence of perceived stigma, its association with socio-demographic variables and its effect on work and social roles limitations among Saudis. These findings can help professionals and policy makers to better understand the effect of perceived stigma on seeking treatment. Consequently, better and focused awareness campaigns can be designed to encourage people to seek help confidently.

The experiment was not preregistered; Deidentified data for experiment along with a codebook and the data-analysis scripts are not posted but can be requested from the principal investigator directly through email; access to the data is limited to qualified researchers.

## Methods

### Sample

The SNMHS was based on a multi-stage clustered probability sampling representative to Saudi Arabia, as detailed elsewhere The SNMHS is a large survey study using a complex, weighted survey design. Several published papers discuss the study design and methodology, including the weighting procedure, in detail. Briefly, several weights were developed for the SNMHS to enable estimation and inference for several types of populations. We cited and highlighted key methodological components, including the weighting procedures. The paper cited below includes all formulas needed for calculation of weights and replication of analyses [31, 32]. Households of Arabic-speaking residents aged between 15 and 65 years old were approached. This age range selected similar to other countries that are part of the World Health Organization’s World Mental Health Survey Initiative. This helps in pursuing accurate cross-national comparisons. Face-to-face interviews were administered by trained interviewers, and carried out between 2011 and 2016. Having sensitive sections in the interview required the respondents to answer the questions individually and privately using laptops. The survey was administered in two parts. Part I was the core diagnostic assessment (*n* = 4004); part II included questions about risk factors, consequences and other correlated assessments and additional mental disorders. Part II was administered to respondents of part I who were diagnosed with a mental disorder plus 25% probability sub-sample of the others (*n* = 1981).

**Fig. 1:**
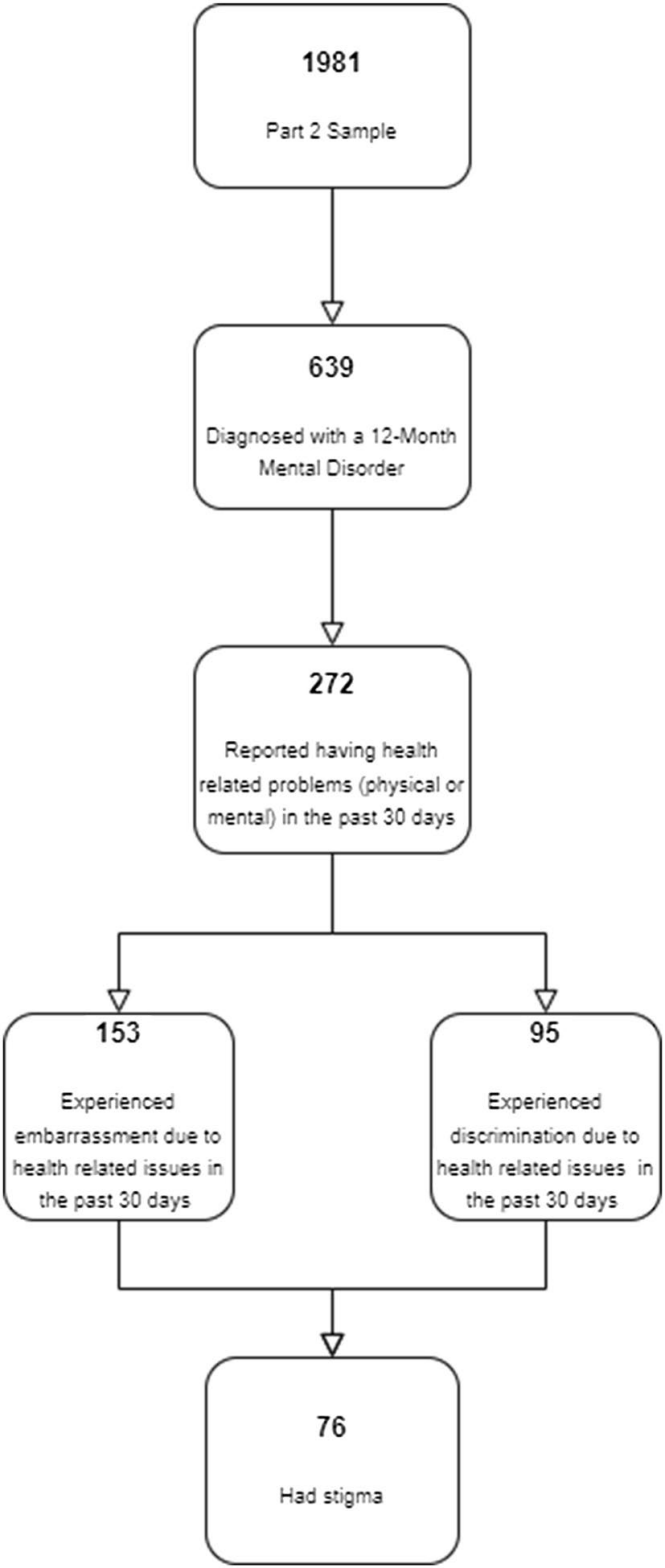
Study sample flowchart

Details of quality assurance and quality control procedures were published elsewhere [33]. Informed consent from the participants was taken before each interview and the study protocol was approved by the Institutional Review Board at King Faisal Specialist Hospital and Research Centre, Riyadh. A total of 1981 respondents who completed the part II diagnostic survey; 639 (32.3%) were diagnosed with a mental disorder and among them, 273 (42.7%) were asked the two questions about discrimination and embarrassment Fig. 1.

### Diagnostic assessment

Psychiatric diagnosis was based on the validated WMH-Composite International Diagnostic Interview (CIDI) 3.0 [34]. This is a fully structured interview that in the SNMHS made assessments using the DSM-IV criteria. The computerized version (CAPI) of CIDI 3.0 translated and culturally adapted based on standardized protocol to create the Arabic version [27], followed by linguistic validation and cultural adaptation [31, 35]. Mental disorders in the CIDI instrument were grouped as follows: mood disorders (major depressive disorder [MDE], bipolar I and II disorder), anxiety disorders (panic disorder, agoraphobia, social phobia and post-traumatic stress disorder), impulse control disorders (conduct disorder, attention-deficit/hyperactivity disorder, intermittent explosive disorder), eating disorders (bulimia, anorexia nervosa and binge eating disorder) and substance use disorders (alcohol and drug abuse and dependence).

### Perceived stigma

Two dimensions were assessed for perceived stigma in our study: embarrassment and perceived discrimination. We used these two items included in the Part II interview that are derived from the European Study of the Epidemiology of Mental Disorders ESEMeD version of the World Health Organization Disability Assessment Schedule II WHODAS-II questionnaire [8], as a measure of function and disability in the 30 days prior to the interview. The Arabic version of these two questions was tested and culturally adapted by experts before use. The question related to embarrassment was: “How much embarrassment did you experience because of your mental health problems during the past 30 days?” and discrimination: “How much discrimination or unfair treatment did you experience because of your mental health problems during the 30 days?”. The response options to both questions were “None/a little/some/a lot/or extreme”. We considered stigma when the two dimensions were present (i.e., if the respondent reported at least “a little” embarrassment and “a little” discrimination). Alonso et al. published this method to evaluate perceived stigma. Although not validated, we used it here since it is part of the CIDI survey and provides information on two key concepts of self-stigma.

Because perceived stigma related to health problems is most relevant to persons with significant health problems, the stigma questions were only administered to individuals diagnosed with 12-month mental disorders who reported significant activity limitation due to health problems in the month prior to the interview (*N* = 273). This approach was adopted previously in Alonso J. et al. study [8].

### Outcome variables

Social limitation, work/role limitation, and treatment seeking were used to assess the impact of perceived stigma. Work and role limitation in the month before the interview was assessed by the questions taken from the WHODAS-II [36]. A Work Lost Days (WLD) index was obtained by the weighted sum of: (A) the number of days totally unable to work or carry out normal activities in the prior month, (B) one half the number of days of reduced activities, (C) one quarter the number of days requiring extreme effort to perform the usual activities [8]. If the sum was more than 30, it was recorded as equal to 30 so that the sum ranges from 0 to 30. The scores were linearly transformed to a 0–100 range, to facilitate interpretation. Social limitation index during the prior 30 days was also assessed using questions taken from WHODAS-II [37]. It was computed by dividing the number of days with difficulties to talk, meet and or socialize with family and friends by 30 and multiplied by 100. Respondents who didn’t record any difficulties reported 0 days. The range of the index was between 0 and 100, where the higher the score meant higher level of social limitation.

### Other variables

This study measured sociodemographic variables extracted for all respondents who were diagnosed with a 12-month mental health condition. These included gender (male/female), age (15–18 years, 19–34 years, 35–49 years, and 50–65 years), completed years of education (0–6 years i.e., low, 7–9 years i.e., low average, 10–15 years i.e., high average, and 16 years or more i.e., high), income (low, low average, high average, high), marital status (married, currently unmarried and never married) and employment (employed, currently unemployed, student). Finally, urbanicity and region were extracted from the sample frame that was provided by the General Authority for Statistics [38] in Saudi Arabia.

Other variables that were investigated included questions that asked whether respondents sought any type of treatment during the past 12 months and if they were aware of the existence of a mental disorder.

### Statistical analysis

Descriptive and bivariate analysis was done using independent chi-square and student t-test, where applicable. A multinomial logistic regression model was generated to calculate the odds ratios (OR), 95% confidence intervals (CI); and interaction effects to assess the effect of the socio-demographic variables in the perceived stigma group. A multivariate linear regression was applied to assess the work/role and social limitations as outcomes, after controlling for several socio-demographic variables: including age, gender, marital status, education and outcome. All analyses used weighted data to adjust for sampling and stratification probabilities as well as to ensure the representativeness of the sample [32].

Data were analyzed using statistical analysis software SAS 9.2 (SAS Institute, Cary, NC), with the PROC FREQ, PROC SURVEYFREQ and PROC LOGISTIC procedures. Statistical significance identified using the conventional threshold of p-value less than 0.05.

## Results

We had data from 272 respondents who reported having health related problems (physical or mental) in the last* 30 days* and answered the two questions about discrimination and embarrassment. Out of these 272 respondents, 153 (56%) reported an embarrassment experience and 95 (34.8%) reported a discrimination experience. When both dimensions were reported, the perceived stigma was observed in 76 (27.8%) individuals with mental illness.

Perceived stigma was significantly more prevalent among females than in males (74.5% vs. 25.6%, *P* = 0.029). There was no significance observed among different age groups. There was no significant difference found among individuals with high average education compared to low average education (49% vs. 15.5%, *P* = 0.93), currently unemployed compared to employed (50.9% vs. 27.3%, *P* = 0.36), married compared to unmarried (49.9% vs. 6.27%, *P* = 0.10), and those with low income versus high income (43.2% vs. 26.7%, *P* = 0.45). Among all who reported experience of perceived stigma, 89% had been diagnosed by the survey with mental illness only while 10.9% had comorbid physical conditions in addition to the mental issues. The majority (90.1%) had no sufficient awareness of having any mental disorders, and didn’t seek any type of treatment in the past 12 months (81.4% vs. 18.5%, *P* = 0. 0.1802) (Table 1).

**Table 1:** Sample characteristics *N* = 272

	Embarrassment		Discrimination		Stigma	
	*N* = 153		*N* = 95		*N* = 76	
	*N*	Weighted %	*N*	Weighted %	*N*	Weighted %
Age						
15–24	42	33.62	32	40.93	23	39.15
25–34	42	21.39	27	27.90	23	24.68
35–49	50	31.99	30	24.68	25	29.22
50–65	19	13.00	6	6.49	5	6.95
Gender						
Male	34	20.58	28	36.44	22	25.46
Female	119	79.42	67	63.56	54	74.54
Education						
Low	25	18.18	14	12.81	12	14.64
Low average	23	17.90	9	11.01	9	15.54
High average	76	39.93	57	59.65	42	49.01
High	29	23.99	15	16.53	13	20.81
Urban/Rural						
Rural	14	7.57	6	3.77	6	5.32
Urban	139	92.43	89	96.23	70	94.68
Region						
Central	60	34.05	33	25.82	23	23.63
Eastern	21	18.64	12	18.97	11	16.69
Northern	16	6.48	10	7.94	9	11.09
Southern	11	9.25	7	11.58	4	5.96
Western	45	31.58	33	35.68	29	42.64
Income						
Low	68	41.02	46	41.28	38	43.66
Low average	19	11.89	14	16.85	10	17.34
High average	27	18.88	11	10.32	10	14.44
High	39	28.21	24	31.56	18	24.56
Mental disorder						
Only mental disorder	143	89.07	90	91.31	73	93.28
Mental disorder and CC	10	10.93	5	8.69	3	6.72
Awareness of mental disorder						
Aware of mental disorder	16	9.85	11	17.22	9	16.30
Not aware	83	90.15	48	82.78	36	83.70
Missing frequency	54		36		31	
Reasons for not getting treatment (concerned about other people)						
Concerned	9	48.45	7	43.04	5	46.50
Not concerned	14	51.55	9	56.96	7	53.50
Missing frequency	130		79		64	
Treatment						
No treatment	114	81.41	66	74.26	52	73.27
Any treatment	39	18.59	29	25.74	24	26.73
Employment status						
Employed	54	36.63	32	32.74	28	42.03
Currently unemployed	62	41.91	39	28.89	31	31.89
Student	36	21.45	24	38.37	17	26.08
Missing frequency	1					
Ethnicity						
Arab	153	100	95	100	76	100
Non-Arab	0		0		0	
Marital status						
Married	94	49.94	51	39.04	45	49.95
Currently unmarried	15	15.18	10	10.37	6	6.27
Never married	44	34.88	34	50.60	25	43.79

Part II weights were used

We had data from 272 respondents who met our inclusion criteria. Out of these 272 respondents, 153 (56%) reported an embarrassment experience and 95 (34.8%) reported a discrimination experience. When both dimensions were reported, the perceived stigma was observed in 76 (27.8%) individuals with mental illness

Stigma = People who reported both Embarassment(FD17) and Discrimination(FD18)

Mental disorder: yes, contains only the people diagnosed with 12-month disorders (people diagnosed with a disorder and no CC conditions)

Mental disorder: comorbid physical conditions, contains people diagnosed with both a 12-month disorder and a chronic condition

Awareness of mental disorder was measured using SR16 (those that seeked professional help) and SR112 (those that did not want to seek professional help)

Reasons for not getting treatment was measured using SR116h and SR126g

Employment status: employed includes self-employed & others

Employment status: currently unemployed includes looking for work, temporarily laid off, retired, homemaker, disabled & don't know

Marital status: currently unmarried includes separated, divorced & widowed

When adjusting for all demographic variables in a logistic regression model, perceived stigma was not significantly associated with any of the variables (see Table 2). We analyzed whether the type of mental disorder was associated with presence of stigma compared to other types; although no significant relationship had appeared to us, among those who reported perceived stigma 31% were diagnosed with social phobia, and 42% were drug independent. (Table 3).

**Table 2:** Multivariate logistic regression analysis of all variables

	Stigma	
	OR	(95% CI)
Age		
15–24	4.41	(0.46–42.66)
25–34	1.63	(0.22–11.84)
35–49	2.37	(0.38–14.9)
50–65	1.00	
Wald χ^2^	2.76	
Gender		
Female	2.22	(0.76–6.51)
Male	1.00	
Wald χ^2^	2.10	
Education		
Low	0.87	(0.16–4.67)
Low average	0.66	(0.14–3.16)
High average	0.63	(0.17–2.39)
High	1.00	
Wald χ^2^	0.56	
Urban/Rural		
Rural	0.63	(0.09–4.73)
Urban Ψ	1.00	
Wald χ^2^	0.20	
Region		
Central	0.31	(0.1–0.93)
Eastern	0.74	(0.2–2.72)
Northern	4.05	(0.61–27.01)
Southern	0.38	(0.06–2.3)
Western	1.00	
Wald χ^2^	8.87	
Income		
Low	1.07	(0.31–3.74)
Low average	1.30	(0.32–5.3)
High average	0.33	(0.07–1.48)
High	1.00	
Wald χ^2^	3.36	
Mental disorder		
Only mental disorder	1.00	
Mental disorder and CC	0.30	(0.06–1.55)
Wald χ^2^	2.07	
Treatment		
No treatment	0.34	(0.1–1.11)
Any treatment	1.00	
Wald χ^2^	3.22	
Employment		
Employed	1.09	(0.34–3.49)
Currently unemployed	0.53	(0.16–1.69)
Student	1.00	
Wald χ^2^	2.11	
Marital status		
Married	1.00	
Currently unmarried	1.76	(0.43–7.24)
Never married	0.35	(0.04–3.12)
Wald χ2	3.62	

Part II weights were used (*N* = 272)

^*^p < 0.05. **p < 0.01

Mental disorder: yes, contains only the people diagnosed with 12-month disorders (people diagnosed with a disorder and no CC conditions)

Mental disorder: comorbid physical conditions, contains people diagnosed with both a 12-month disorder and a chronic condition

Employment status: employed includes self-employed & others

Employment status: currently unemployed includes looking for work, temporarily laid off, retired, homemaker, disabled & don't know

Marital status: currently unmarried includes separated, divorced & widowed

**Table 3:** The distribution of mental health illness in the population and its correlation with stigma

Disorder	Stigma				
	*N*	%	SE	*χ*^2^	*P*-Value
Anxiety disorder					
Panic disorder^1^	7	27.71	10.94	0.4252	0.5149
Generalized anxiety disorder^1^	2	4.65	3.71	3.3438	0.0686
Social phobia^1^	20	31.36	8.22	2.3203	0.1289
Agoraphobia ^1^	10	29.64	9.12	0.9914	0.3203
Post-traumatic stress disorder^2^	12	19.28	7.03	0.0092	0.9238
Adult separation anxiety disorder^2^	21	24.64	7.08	0.6749	0.4121
Obsessive–compulsive disorder^2^	12	25.52	8.59	0.5223	0.4705
Any anxiety disorder^2^	46	20.64	3.82	0.1117	0.7384
Mood disorder					
Major depressive disorder^1^	14	23.18	7.82	0.1146	0.7097
Bipolar I and/or II^1^	15	20.73	7.56	0.0008	0.9776
Any mood disorder^1^	29	22.01	5.50	0.0803	0.7771
Impulse disorder					
Conduct disorder^2^	4	59.25	21.81	1.5047	0.2210
Attention deficit disorder^2^	22	24.55	6.89	0.6787	0.4108
Intermittent explosive disorder^2^	11	20.69	7.79	0.0113	0.9154
Any impulse-control disorder^2^	30	23.30	5.49	0.6396	0.4246
Substance disorder					
Alcohol abuse^2^	0	–	–	–	–
Alcohol dependence^2^	0	–	–	–	–
Drug abuse^2^	14	27.34	12.03	0.6039	0.4378
Drug dependence^2^	5	42.79	22.67	1.5027	0.2213
Any substance use disorder^2^	15	23.79	9.31	0.2382	0.6259
Eating disorder					
Anorexia^2^	0	–	–	–	–
Binge eating disorder^2^	11	20.41	8.48	0.0035	0.9528
Bulimia^2^	4	15.06	8.94	0.2446	0.6213
Any eating disorder^2^	15	19.44	7.08	0.0058	0.9392
Any^2^	76	19.95	2.91	–	–

^1^Part1 sample, prevalence calculated using part 1 weights

^2^Part2 sample, prevalence calculated using part 2 weights

*N* = 272

^*^p < 0.05. **p < 0.01

When we compared the scores of work role and social limitations outcomes in respondents who reported perceived stigma versus those who didn't, we found that the scores were higher among respondents who reported stigma. The scores of the work role limitation were 10.5 vs. 7.9 while social limitations were 2.9 vs. 2.4. Individuals who reported stigma had higher work/role limitations OR = 1.1 (95% CI 1.06–0.1017, *P* 0.006) and higher social limitations OR = 1.3 (95% CI 0.99–1.62, *P* 0.05) compared to individuals who didn’t report stigma (Tables 4, 5).

**Table 4:** Means of work lost days and social limitation in the comparisons groups

	Work lost days		Social limitation	
	*N*	Mean	*N*	Mean
Stigma	126	10.55	38	2.95
No stigma	408	7.91	93	2.45

**Table 5:** Effect size of the stigma on the work lost days and social limitation

	OR	(95% CI)	Wald Chi-Square	Pr > ChiSq
Work lost days	1.04	(1.01–1.07)	5.46	0.02*
Social limitations	1.28	(1.00–1.63)	3.83	0.05*

^*^p < 0.05. **p < 0.01

## Discussion

Our study shows that perceived stigma is prevalent (27.8%) among Saudi individuals who have been diagnosed with a mental illness in the past 12 months. Perceived stigma was not associated with any of the demographic variables, however, it was associated with work and role limitations, and the social limitations of the individuals. To our knowledge, this is the first national, population-based survey to assess perceived stigma related to mental illnesses among Saudi respondents and its effect on their social life and work.

We identified perceived stigma based on the effect of two indicators: embarrassment and discrimination consistent with previous studies [8]. Although this approach might underestimate the prevalence of stigma, the rational for adopting this classification was due to limited understanding of stigma in Arab countries which makes it serve as a reasonable measurement especially when the following points are considered [39].

It seems that respondents with mental illness tend to decide on their own or follow the decision of a caregiver to isolate from the society, which limits their experience of recognizing embarrassment and discrimination behavior outside their closed framework of society [40]. The high proportion 153/273 (56%) of people who experienced embarrassment in our study emphasizes that self-stigma might be behind these beliefs. Moreover, it has been well documented that those who report mental illness stigma are more likely to have low self-esteem [41]. Some individuals with mental illness might also have beliefs not related to clinical issues, like lack of faith in God, evil eye *“hasad”* [42] or black magic “*Sehr”* [30]; these can play a role in the lack of understanding and the presence of mental illness related-stigma. Overall, lack of proper understanding, adequate mental health services distribution, service quality and poor literacy about mental illnesses might be some reasons for 83% of the study’s respondents not being aware of their mental illness. This finding is consistent with a study from KSA where 87.9% of the general population showed poor knowledge about psychiatric disorders. Additionally, 47% of the general population did not want people to know about their mental illness and 20.7% admitted being ashamed if a family member had mental illness, which further described their stigma-related attitudes towards detachment and isolation from the surrounding society [40]. Our findings also align with the INDIGO network survey where DISC-12 scale was used to interview 729 people with a clinical diagnosis of schizophrenia across 27 countries, findings that over 90% had experienced discrimination due to their mental health status. Most people (72%) reported a need to conceal their diagnosis [22].

Compared to other countries like Brazil which reported prevalence of perceived stigma as 14.8% [8], and European countries that reported self-stigma as 41% [23], our estimate (27.8%) falls in the middle of that range. However, there are other studies from Saudi Arabia that previously examined stigma and reported higher stigma-related rates from the public’s point of view [30]. For example, in one Saudi study, 66% of the general population showed negative perception and negative attitude towards mental illness [40]. However, these questions were not direct and might not accurately represent how individuals with mental illness feel about others' perceptions towards them.

Although perceived stigma was higher among patients who didn’t sought treatment during the last 12 months (73.2% vs 26%), however, it wasn’t statistically significant compared to those who did. Perceived stigma might be a minor reason for not seeking treatment as reported by another study from SNMH data, where it was found that only 4.7% out of 309 participant reported stigma as an attitudinal barrier [28].

Unlike several studies that found stigma to be a significant barrier for seeking treatment [10, 43, 44]. However, in most studies on this subject, data on stigma were collected from the general population and not from individuals with mental illness specifically [45]. There are many pathways at the micro- and macro-level through which stigma can manifest then have implication on seeking help. As cited earlier the misclassification of mental illness from the society may lead to discrimination, stereotype and rejection from the normal society engagement. That would discourage patients from asking help as they assume that they would be labeled as mentally ill with limited privileges [46]. In Saudi Arabia, this attitude appears more with the female patient, where she tends to hide her illness as a fear of being socially disqualified. Also female patients tend to avoid seeking help of a male doctor due to a reluctance to disclose personal information to male strangers. Another point that patients—regardless the gender—may not trust the medical care facilities for their confidentiality, they might think that their information about the mental illness will be shared with employers which might directly affect their job [47]. This might explain why females with mental illness and didn’t seek treatment in our study reported more perceived stigma compared to those who sought treatment.

Our data also showed that half of the respondents who reported perceived stigma were currently unemployed. We found a significant relationship after modeling work limitation and social limitation with stigma. People who reported stigma were more likely to have their work and social life affected. This finding is consistent with other studies worldwide [4, 48]. Brohan et al. when used (DISCUS) found that the most common experiences of discrimination were being shunned at work and discrimination in making or keeping friends [49]. The reasons for this could be that employers tend to deter from hiring and retaining mental health patients because of their cognitive and emotional limitations, which cause reduced productivity and increased costs of disability [50].

## Limitations

The results of this study must be interpreted while considering some limitations. First, approximately 40% of the sample that was diagnosed with 12-month mental disorder was asked the two specified questions related to stigma. Those who were not asked these two questions might have different perspectives. They may have different responses to the stigma questions. Second, not using a validated survey specific for perceived stigma “self-stigma” caused that other indicators (like coping orientation, or rejection) [24] were not assessed in this study. This may imply that our estimate might not be comprehensive in assessing perceived stigma. Third, the study was conducted between 2011 and 2016, it’s possible that the recent changes worldwide and the local efforts to increase awareness against mental health stigma might reflect different figures of stigma. However, probably it will take more time to actually see the change in the practice and the perceptions of the public towards this issue. Finally, these data are cross-sectional in nature and, therefore, it is not possible to infer causality between perceived stigma and work/social-related outcomes.

The findings of our study indicated that individuals who had been diagnosed with a mental illness within the last year encountered a significant amount of stigma. It is crucial to emphasize the practical implications of these results. To address this issue, it is important to educate healthcare providers on the importance of avoiding stigmatizing behaviors and ensuring the confidentiality of patients with mental illness. Additionally, there is an urgent need to raise public awareness and discourage stigmatizing attitudes, particularly among employers and educators [48, 50]. This can be achieved by promoting acceptance and implementing specific accommodations for individuals with mental illness in the workplace. It is worth noting that further longitudinal research is necessary to validate our findings and identify timely risk factors associated with stigma.

## Data Availability

The datasets used and analyzed during the current study are available from the corresponding author on reasonable request.

## Abbreviations

DSM-IV: Diagnostic and Statistical Manual of Mental Disorders
CIDI: Composite International Diagnostic Interview
SNMHS: Saudi National Mental Health Survey
